# Gut-brain axis: altered microbiome and depression – review

**DOI:** 10.1097/MS9.0000000000000573

**Published:** 2023-04-13

**Authors:** Anmol Mohan, Swathi Godugu, Saumya S. Joshi, Kenisha B. Shah, Srija C. Vanka, Hania Shakil, Dhanush P, Swathi Veliginti, Prajwal S. Sure, Jyothsna Goranti

**Affiliations:** a Department of Medicine, Karachi Medical & Dental College, Karachi, Pakistan; b Department of Medicine, Zaporozhye State Medical University, Zaporozhye, Ukraine; c Department of Medicine, Gujarat Medical Education and Research Society, Sola, Ahmedabad; d Dr Pinnamaneni Siddhartha Institute of Medical Sciences and Research Foundation, Chinna Avutapalli, Andhra Pradesh; e Department of Medicine, Bhaskara Medical College, Telangana; f Department of Medicine, Kempegowda Institute of Medical Sciences, Bangalore, Karnataka; g Department of Internal Medicine, Hackensack Meridian Health-Palisades Medical Center, New Jersey, USA; h Hamdard College of Medicine and Dentistry, Hamdard University, Pakistan Medical Commission, Karachi, Pakistan

**Keywords:** depression, gut microbiota, gut-brain axis

## Abstract

The concept of a ‘gut-brain axis’ was recently developed when the complex communications between the brain and the gut became evident. The interaction may affect emotions, motivation, mood, and higher cognitive functions as well as gut homeostasis. Human microbe symbiosis’s merits are now acknowledged to transcend human mental health. Research has recently indicated that the gut-brain axis plays a vital role in brain health maintenance. The term ‘gut-brain axis’ can only partially capture the intricacy of these interactions. Dysbiosis of the gut commensals has been seen in patients with psychiatric diseases, such as depression. Major depressive disorder is caused by complicated interactions between the individual gene and the environment. In a forced swimming test, P. Zheng *et al.* discovered that germ-free mice with no gut microbiota had a shorter immobility duration than healthy mice. More radical effects were expressed on the use of probiotics rather than prebiotics and postbiotics in reducing the symptoms of depression in patients with major depressive disorder. One of prime importance can be given to exploring more microbiota to investigate the better therapeutic effects of probiotics, prebiotics, and postbiotics.

## Introduction

Highlights

- Insights into the gut-brain interaction have shown a complex communication mechanism that is believed to affect emotion, motivation, and higher cognitive functions and ensure normal gastrointestinal homeostasis.
- The substantial evidence of alteration in the microbiota explains depressive symptoms in human trials.
- More radical effects were expressed on the use of probiotics rather than prebiotics and postbiotics in reducing the symptoms of depression in patients with major depressive disorder.
- One of prime importance can be given to exploring more microbiota to investigate the better therapeutic effects of probiotics, prebiotics, and postbiotics.

Insights into the gut-brain interaction have shown a complex communication mechanism that is believed to affect emotion, motivation, and higher cognitive functions and ensure normal gastrointestinal homeostasis. The concept of a ‘gut-brain axis’ was recently developed when the complex communications between the brain and the gut became evident. The interaction may affect emotions, motivation, mood, and higher cognitive functions, as well as gut homeostasis. Human microbe symbiosis’s merits are now acknowledged to transcend human mental health. Research has recently indicated that the gut-brain axis plays a vital role in brain health maintenance^1^. The term ‘gut-brain axis’ can only partially capture the intricacy of these interactions^2^.

The gut microbiome serves a variety of purposes. Food fibers and endogenous intestinal mucus are examples of nondigestible substrates that must be fermented by bacteria. This fermentation encourages the survival and growth of bacteria that produce/secrete a wide variety of metabolites, including short-chain fatty acids (SCFAs). The most common SCFAs produced are acetate, propionate, and butyrate^3^. The effects of SCFAs on gut homeostasis range from reducing the risk of colorectal cancer to preserving intestinal barrier integrity, mucus production, and protection against inflammation. The autonomic nervous system (ANS) is thought to govern the function of the gut and the composition of the microflora colony through influencing bowel movements, gut motility, secretion, and gut permeability^4^. The vagus nerve plays a crucial role in communication between the gut and the brain^5^. The gut provides roughly 90% of the input of the N vagus to the brain. This input’s effect on the brain’s function is not fully understood. The role in disease becomes clearer, especially in diseases like depression.

Depression is a mood ailment marked by a continuous sense of melancholy, a loss of concentration, and a loss of interest. According to the WHO, depression is the fourth-largest cause of death among people between the ages of 15 and 29 and results in over 700 000 suicide attempts annually. A more sedentary lifestyle and less exercise have been brought on by changes in work culture, the increased use of electronics, and the internet, both of which are linked to the pathophysiology of depression^6^.

The ANS is thought to govern the function of the gut and the composition of the microflora colony through influencing bowel movements, gut motility, secretion, and gut permeability^4^. The vagus nerve plays a crucial role in communication between the gut and the brain^5^ The role of the microbiome, and in particular, changes in the microbiome for the pathogenesis of depression is not straightforward.

Dysbiosis is described as a change in the local distribution of the microbiota, a change in their functional composition and metabolic processes, or an imbalance in the microflora that upsets the microbiota’s normal balance^7^. Progressively, the etiology of intestinal and extraintestinal disorders is being linked to dysbiosis of the gut flora. Allergies, asthma, metabolic syndrome, cardiovascular disease, and obesity are extraintestinal disorders, whereas irritable bowel syndrome, celiac disease, and inflammatory bowel disease (IBD) are intestinal illnesses. These days, there is a lot of discussion about depression risk, gut inflammation, and obesity. According to one study, teenagers who were obese had a 40% higher risk of being depressed, whereas those who were sad had a 70% higher risk of being obese^8^. Changes in the microbiome play a role in producing proinflammatory cytokines linked to psychiatric illnesses^9^.

In the study, the properties of Lactobacillus Plantarum 299v (LP299v) were examined. Individuals were either given a placebo during the 8-week study or a combination of the probiotic LP299v and a selective serotonin reuptake inhibitor. In neither the probiotic group nor the placebo group did the levels of interleukin (IL)-6, IL-1b, tumor necrosis factor-, or cortisol alter significantly. However, when the LP299v group was contrasted to the placebo group, there was a significant decrease in kynurenine concentration and an improvement in cognitive performance, showing the efficacy of adding particular strains of probiotic bacteria to standard depression therapy^10^.

In this review, we will discuss the evidence for the role of the gut-brain axis in the pathogenesis and/or treatment of patients with depression.

## Pathophysiology of depressive disorder

### Dopamine system

Dopamine is a catecholaminergic neurotransmitter with important signaling pathways in the peripheral and central nervous systems (CNSs), which are linked to significant physiological and pathological events^11^. Dysfunction of dopaminergic signaling has been linked to several cognitive diseases, including Attention Deficit Disorder Attention/Deficit Hyperactivity Disorder, autism, and schizophrenia^12^. Current medical treatments for depression take weeks to achieve full efficacy, are unsuccessful in many individuals, or create unpleasant side effects, suggesting a need for a better knowledge of depressed mood and therapy^13^. Emerging facts have additionally connected dopamine system disorder to the pathophysiology of depression. However, among the signs and symptoms observed in depression, along with anhedonia and apathy, had been more or less continuously related to dysfunctions in the DA system^14^. Antidepressants work by boosting monoamine neurotransmitters (serotonin, norepinephrine, and/or dopamine) within the synaptic cleft. Physiological or emotional stress, and the anxiety it causes, permits an organism to avoid dangers while also providing an incentive to achieve goals.

### Altered stress response

Most studies showing a link between stress and depressive episodes have been based on episodic stressors (one-time occurrences with a beginning and an end) of an unfavorable or unpleasant nature^15^. The evidence to date suggests that the human microbiome is similarly involved in prenatal and early-life stress, with high-stress microbiota containing a greater diversity of Clostridium genera, typically associated with inflammation and illness. However, more human studies are still required in this area^16^. The ANS is another crucial component of the stress response. Heart rate variability can be used to evaluate the imbalance between the sympathetic and parasympathetic neural systems in patients with Major depressive disorder (MDD). The advent of common psychiatric conditions, including post-traumatic stress disorder (PTSD), depression, drug abuse, and schizophrenia, on the other hand, can be a negative effect of more stress^14^.

### Inflammation

A higher risk of depression has been shown with a variety of disorders linked to immune system activation, including allergies, autoimmune diseases, and infections. A part of MDD has been reclassified as a chronic inflammatory illness, similar to diabetes and coronary heart disease, in light of mounting evidence, and the involvement of proinflammatory cytokines like IL-6, IL-1, and tumor necrosis factor in the pathogenesis of the disease^17^. Early studies of the immune system’s relationship to psychological reactions were considered in the context of cytokine-induced illness behavior. Immunotherapies like interferon alpha in the treatment of hepatitis C^18^. Researchers are looking into the impact of peripheral inflammation on the CNS because of the significance of inflammation in depression and weariness. The blood-brain barrier, which isolates the CNS parenchyma from the peripheral blood supply, undergoes many changes^19^. Using a 13C-tryptophan breath test, we showed that the tryptophan-kynurenine pathway is elevatedly activated in MDD patients^20^. Additionally, we discovered decreased plasma levels of tryptophan in MDD patients^21^, which we think is at least partially attributable to the enhanced tryptophan-kynurenine pathway. Kynurenine is converted in the brain to quinolinic acid or 3-hydroxykynurenine, which is thought to play a role in the etiology of depression and has neurotoxic effects as an N-methyl-D-aspartate receptor agonist^21^.

### Brain-derived neurotrophic factor (BDNF)

BDNF is best studied in the hippocampus, where its elevation by antidepressant medications appears critical for behavioral activity^22^. Preclinical research studies have found that BDNF regulates BDNF expression and has antidepressant-like effects in animal studies, implying a function for BDNF in depression. In rodents with a learned helplessness model of depression, BDNF infusion lowers escape latencies and failure rates^23^. Although BDNF levels in the human CSF tend to be undetectable, some studies have detected lower amounts of BDNF propeptide formed during the conversion of pro-BDNF to mature BDNF, in MDD patients compared to healthy controls^24^.

## Gut microbiome and depression

Previously, it was thought that the brain was in charge of all bodily activities. Recent statistics and studies, on the other hand, suggest otherwise. Any alteration in the gut microbiome leads to microbial lipopolysaccharides being produced, which triggers our body’s inflammatory responses. The production of proinflammatory cytokines activates the vagal nerve’s afferent loop, which stimulates the hypothalamic-pituitary-adrenal (HPA) axis and causes psychiatric consequences. On the other hand, some believe that inflammation in the gut causes neuroinflammation, which leads to the kynurenine pathway being activated. All these elements play a role in depression^25^ The substantial evidence of alteration in the microbiota explains depressive symptoms in human trials^26^.

Studies have found a direct correlation between an altered gut microbiome and psychiatric illnesses. Research has pointed out a specific microbe alteration to mental illnesses^27^. Many studies have focused on figuring out how the gut microbiota interacts with the brain. Supplemental probiotics have demonstrated potential results in the treatment of brain-related issues. Although promising, further exploration is needed to determine probiotics’ action mechanisms and negative effects^28^. In stressful situations, the gut microbiota can communicate with the CNS via various channels, including neuroimmune, sensory-neural, and others. It has been discovered that these bacterial commensals can affect stress levels and that stressful experiences can control our gut microbiome makeup.

Dysbiosis of the gut commensals has been seen in patients with psychiatric diseases, such as depression^29^. MDD is caused by complicated interactions between the individual gene and the environment. In a forced swimming test, Zheng *et al.* 30 discovered that germ-free mice with no gut microbiota had a shorter immobility duration than healthy mice. Furthermore, the clinical sample revealed a substantial difference in the makeup of gut commensals between MDD patients and healthy controls. This describes how the gut bacterial environment’s alterations influence the host’s behavior. Recent research has connected neurobiological alterations with the onset of depression. Inflammation is one of the factors that contributes to the modifying process. Inflammation was found to have high levels of neurotransmitter concentration and an increase in psychological processes. The host needs to maintain a stable microbial community to regulate physiology, and it is reasonable to assume that these microbiomes are responsible for depression in a way^31^. Infectious compounds, cytokines, medications, and vagal sensory fibers are a few examples that convey messages to the brain about the status of the stomach. The HPA axis also metabolizes microbiome diversity and nutritional ability^32^.

Inflammatory illness patients have been proven to have depressive symptoms in studies. Depressive symptoms occurring more frequently are a marker of deteriorating IBD. This disorder is thought to be caused by malfunctioning circuits in the gut-brain axis^33^. It is critical to completely comprehend that in order to break the vicious cycle of inflammation resulting in depressive disorders and depression exacerbating cytokine responses, it is necessary to understand the mechanism of inflammation leading to depression and depression worsening cytokine responses^34^. Long-term neuroinflammation has been found to disrupt brain functioning, which could influence an individual’s mood and behavior^35^. Both depression and inflammation interact in a vicious cycle that fuels each other. Inflammation is one of the most essential variables in the occurrence of depression. As a result, it produces more cytokines in reaction to stimuli that are not natural to the human body. The inflammatory response is heightened when several elements, such as genetic factors, infectious diseases, and environmental stressors come together. Depression and other hazardous habits, such as bad food and a sedentary lifestyle may cause an uncontrollable inflammatory response, worsening depression. Stressful experiences influence the diversity and content of an individual’s gut commensals. Inflammation that is recurrent, massive, and chronic damages mental and physical disorders. When a patient suffers from both inflammation and depression, it is vital to address both issues to find a solution^36^.

Depression is becoming more common among IBD patients, affecting the well-being of those who are affected. Immune-inflammatory, oxidative, and nitrosative stress pathways are all involved in both diseases. This is indicated by higher levels of proinflammatory cytokines and acute-phase reactants and lower levels of negative acute-phase reactants. Both depression and IBD have overlapping immune-inflammatory, oxidative and nitrosative stress processes, implying a causal link. These procedures are critical in the treatment of either illness^36^.

## Role of Diet in depression

There are multiple mechanisms through which diet affects mental health. They can broadly be categorized into direct and indirect mechanisms. Direct mechanisms influence the gut microbiome, thereby exerting their influence. Indirect mechanisms are using improved physical health.

### Inflammatory diet

‘Dietary Inflammatory Index’ classifies diets into proinflammatory diets and anti-inflammatory diet. Various studies have reported that diets with higher inflammatory potential are linked to depression^37^,^38^. Vegetables, whole grains, olive oil, and fish have less impact on systemic inflammation. At the same time, foods rich in processed meat, refined flour, and sugars are associated with higher inflammation^39^. Knowing the underlying biochemical mechanisms behind this association is crucial, enabling advancements in interventions to treat depression and related mental ailments better.

The Montgomery-Sberg Depression Rating Scale was used in a randomised control experiment (SMILES trial) comprising 166 people to evaluate the effect of food intervention on depression (MADRS). Dietary therapies included goal-setting, motivational interviewing, tailored dietary recommendations, and nutritional counseling support, as well as mindful eating under the direction of a clinical dietitian. The dietary support group (*n*=31) had greater improvement in MADRS score at 12 weeks (*P*<0.001)^40^. The number needed to treat (NNT) was a remarkable 4, which further supports the push for utilizing dietary interventions as a valuable tool for treating depression.

The Mediterranean diet (MedDiet) has been proven beneficial in cardiovascular health, as seen in the landmark PREDIMED study^41^. Adherence to Mediterranean diet has been shown to have a significant impact on mental health as well. The intervention arm saw a 1.68-fold reduction in symptoms of depression^42^.

### Effect of micronutrients

The role of several micronutrients in the development of depression has been assessed in multiple studies. In middle-aged men, magnesium intake was associated with a protective effect against depression^43^. Also, a higher intake of folic acid, vitamin B12, and B6 reduced the risk of depression^44^. Chang *et al.* 45 found a 7–10% lower risk of depression in individuals with increased dietary flavonoid intake, especially in older women. Intake of probiotics and prebiotics has been shown to improve intestinal health, thereby reducing inflammation and the incidence of mental illness^46^.

### Specific food and its role in depression

Consumption of fish or omega-3 fatty acids has been associated with a reduced risk of depression in a dose-response fashion^47^. In contrast, consumption of sugar-sweetened beverages carries a modestly higher risk of depression^48^. The association between vegan/vegetarian diets and depression is controversial. Exclusion of any food group from the diet, regardless of the food type, was associated with increased symptoms of depression^49^.

## The effects of probiotics, prebiotics, and postbiotics on depression symptoms

The use of probiotics, prebiotics, and postbiotics have beneficial effects on the mood and psychological well-being of patients with depression^50^,^51^. The term probiotic has evolved from the word ‘pro bios’, meaning for life, that is, the living organism residing in the healthy individual gastrointestinal tract^50^,^51^. The microorganism comprises various bacteria, viruses, protozoans, and fungi. These organisms feed on certain types of soluble fibers provided as prebiotics SCFAs, often known as probiotics, are created when bacteria ferment soluble fiber and include butyric acid, acetic acid, or propionic acid (by-products). In addition to supporting the immune system, giving the gut bacteria energy, and improving mental health, SCFAs are important for intestinal health^50^,^51^. While also contributes to altered levels of serum cytokines, decreased cortisol, and brain neurotransmitter and protein concentrations, which ultimately result in behavioral changes, as shown in clinical research^51^,^52^.

A randomized control trial including 110 MDD patients was divided into groups, one receiving probiotics (*n*=36), the second one receiving placebo (*n*=38), and the last group receiving prebiotics (*n*=35). The patients were followed up for 8 weeks and, based on BDI scores (*P*=0.01), showed a significant decrease in depression in the probiotic group compared to the placebo and prebiotic group^50^,^51^. A randomized control trial included 40 MDD patients equally divided into two groups, one receiving probiotics and the other receiving a placebo. The subjects followed for up to 90 days and assessed on the (HAMD: *P*=0.005, MADRS: *P*=0.007, CES-D: *P*=0.009) showed a significant decrease in the controlled group rather than in the placebo group^50^,^51^. In a clinical trial, double-blind, placebo-controlled, randomly divided two groups of 60 adult students receiving postbiotics twice daily and placebo were assessed for 24 weeks based on changes in SCFAs concentrations in feces, salivary cortisol levels, and fecal microbiota analysis. The levels were analyzed by Analysis of Covariance, which showed significance in the levels of SCFas and fecal microbiota (*P*<0.05)^51^,^52^.

## Role of the HPA axis in altered microbiome and depression

It has been noted that in depressed patients, activation of the HPA axis can alter the microbiota’s makeup. Neuroendocrine diseases can be caused by altered microbiota composition and increased intestinal permeability. Early-life stress has a major impact on HPA axis activation^53^. Intestinal microorganisms would play a significant role in regulating the HPA axis. The neuroendocrine system in the brain can be impacted by imbalances of the HPA axis brought on by gut bacteria, which can elicit anxiety-like behavioral phenotypes. The recent research raises the possibility that innovative treatments for stress-related illnesses, such as anxiety disorders, may be created by directly or indirectly altering the intestinal microbial flora while using drugs that are already on the market^54^.

## Conclusion

The studies show a promising future with probiotics, prebiotics, and postbiotics on the brain-gut microbiota in patients with MDD. More radical effects were expressed on the use of probiotics rather than prebiotics and postbiotics in reducing the symptoms of depression in patients with MDD. One of prime importance can be given to exploring more microbiota to investigate the better therapeutic effects of probiotics, prebiotics, and postbiotics.

## Ethical approval

Not applicable.

## Consent

Not applicable.

## Sources of funding

None to disclose.

## Author contribution

Conception and design of the study as well as drafting the text is done by all authors.

## Conflicts of interest disclosure

None to disclose.

## Provenance and peer review

Not commissioned, externally peer reviewed.
